# Detection of *bla*_*NDM−1*,_*mcr-1* and *MexB* in multidrug resistant *Pseudomonas aeruginosa* isolated from clinical specimens in a tertiary care hospital of Nepal

**DOI:** 10.1186/s12866-023-02906-w

**Published:** 2023-05-25

**Authors:** Samikshya Sharma, Madhu Dixit Devkota, Bharat Mani Pokhrel, Megha Raj Banjara

**Affiliations:** 1 Central Department of Microbiology, Tribhuvan University, Kirtipur, Kathmandu 44613 Nepal; 2 Upendra Devkota Memorial National Institute of Neurological and Allied Sciences, Bansbari, Kathmandu 44600 Nepal

**Keywords:** *Pseudomonas aeruginosa*, *bla*_*NDM−1*_, *mcr-1*, *MexB*, Multidrug resistant

## Abstract

**Introduction:**

*Pseudomonas aeruginosa* is an opportunistic pathogen, which causes healthcare-associated infections in immunosuppressed patients. They exhibit resistance to multiple classes of antibiotics via various mechanisms such as the over-expression of efflux pumps, decreased production of the outer membrane protein (D2 porin), over-expression of the chromosomally encoded AmpC cephalosporinase, modification of drugs, and mutation(s) at the target site of the drug. The bacteria also develop antibiotic resistance through the acquisition of resistance genes carried on mobile genetic elements. Limited data on phenotypic as well as genotypic characterization of MDR *P. aeruginosa* in Nepal infers the needs for this study. This study was carried out to determine the prevalence rate of metallo-β-lactamase (MBL-producer) as well as colistin resistant multidrug resistant (MDR) *P. aeruginosa* in Nepal and also to detect MBL, colistin resistance, and efflux pump encoding genes i.e. *bla*_*NDM−1*_, *mcr-1 *and *MexB* respectively in MDR *P. aeruginosa* isolated from clinical samples.

**Methods/methodology:**

A total of 36 clinical isolates of *P. aeruginosa* were collected. All bacterial isolates were phenotypically screened for antibiotic susceptibility using Kirby Bauer Disc Diffusion method. All the multidrug resistant *P. aeruginosa* were phenotypically screened for MBL producer by Imipenem-EDTA combined disc diffusion test (CDDT). Similarly, MIC value for colistin was also determined by broth microdilution method. Genes encoding carbapenemase (*bla*_*NDM−1*_), colistin resistant (*mcr-1*) and efflux pump activity (*MexB*) were assayed by PCR.

**Results:**

Among 36 *P. aeruginosa*, 50% were found to be MDR among which 66.7% were found to be MBL producer and 11.2% were found to be colistin resistant. Among MDR *P. aeruginosa*, 16.7%, 11.2% and 94.4% were found to be harbouring *bla*_*NDM−1*_, *mcr-1* and *MexB* genes respectively.

**Conclusion:**

In our study, carbapenemase production (encoded by *bla*_*NDM−1*_), colistin resistant enzyme production (encoded by *mcr-1*), and expression of efflux pump (encoded by *MexB*) are found to be one of the major causes of antibiotic resistance in *P. aeruginosa*. Therefore, periodic phenotypic as well as genotypic study in Nepal on *P. aeruginosa* would provide the scenario of resistance pattern or mechanisms in *P. aeruginosa*. Furthermore, new policies or rules can be implemented in order to control the *P. aeruginosa* infections.

**Supplementary Information:**

The online version contains supplementary material available at 10.1186/s12866-023-02906-w.

## Introduction

*Pseudomonas aeruginosa* is a most common pathogen that causes serious nosocomial and opportunistic infections in immunosuppressed patients [1]. Due to the inherent and acquired mediated resistance to available antibiotics, *P. aeruginosa* is currently turning into the most terrifying pathogen, or “Superbugˈˈ whose last resort of antibiotics is carbapenem [2, 3]. However, resistance to carbapenem is also emerging rapidly nowadays with the prevalence rate between 5 to 25% in Nepal [4]. As a result, by 2018 WHO has listed such carbapenem resistant *P. aeruginosa* under critical group for which no new antibiotics are developed till date [2]. Due to this reason, despite having several side effects (i.e. nephrotoxicity and neurotoxicity effect), an old antibiotic i.e. colistin are now used as a last line drugs to treat such emerging pathogens [2, 5]. However, colistin resistant *P. aeruginosa* is also being reported recently that is in increasing trend globally [6]. Similarly, in *P. aeruginosa* along with carbapenem and colistin resistant genes, overexpression of efflux pump is also one of the intrinsic mechanism that is serving them to develop into superbugs.

In *Pseudomonas* spp., mostly metallo-β-lactamases (MBLs) type carbapenemases are of particular concern because of their rapid spread and sturdy carbapenemase activity [3]. Several types of MBLs such as VIM, SPM, IMP, AIM, GIM, FIM and NDM and their variants have been reported [7]. Among them most common type in *P. aeruginosa* is NDM type; as it is located in plasmid and have a high global dissemination rate [8, 9]. Likewise, in Nepal, the prevalence of colistin resistant *P. aeruginosa* has increased to 2.8% [6]. Resistance to colistin in bacterial species can be either intrinsic type or acquired via chromosomal mutation or genes carried on plasmid [10]. Thus far, only one mechanism that can be transferred through plasmid (i.e. *mcr*) has been detected [11]. Even though other variants of *mcr* (*mcr-2* to *-9*) have been detected, *mcr-1* is the most prevalent marker to date globally [8].

In *P. aeruginosa*, MexAB-OprM is one of the paramount types of efflux pump that is expressed constitutively. It is leading *P. aeruginosa* to develop into multidrug resistant by targeting multiple classes of antibiotics including β-lactam, fluoroquinolones, tetracyclines, chloramphenicol, macrolides, novobiocin, trimethoprim and sulphonamides [9, 12].

Therefore, overexpression of efflux pump, emergence of carbapenem and colistin resistant *P. aeruginosa* are leaving no options for treatment of infection caused by *P. aeruginosa*. If no new antibiotics are developed in future, increase in prevalence of superbugs would be the major cause of high mortality rate in future.

In Nepal, limited studies on carbapenemase producer *P. aeruginosa* are available. It is essential to report present scenario of prevalence of carbapenem resistant *P. aeruginosa*. Similarly, there is no report of *mcr-1* and *MexB* harbouring *P. aeruginosa* in Nepal. Therefore, it is essential to conduct this study to understand the mechanisms of resistance in MDR *P. aeruginosa*. Similarly, this study also helps to understand global spread scenario of plasmid mediated *mcr-1* and *bla*_*NDM−1*_.

## Materials and methods

This study was hospital based cross-sectional study. The phenotypic study was conducted in Microbiology Department of Upendra Devkota Memorial National Institute of Neurological and Allied Sciences (UDM-NINAS), Bansbari, Kathmandu from January to August 2021. Further, the genetic analysis was carried out in Central Department of Microbiology, Tribhuvan University, Kirtipur, Kathmandu.

### Ethical approval and consent from the participant

The ethical approval (Ref. No.: 117/2021) of the study was obtained from the Institutional Review Committee of UDM-NINAS. Written informed consent was obtained from the patients before collection of specimens and data. The data were kept confidential.

### Sample collection and transport

Seven hundred seventy clinical samples including tracheal aspirates, pus, urine, tip of Foley’s catheter, sputum, throat swab, blood, CSF, pleural fluid, CVP tip were collected from all age group of both genders visiting hospital during our study period. All the samples were labelled properly with patient’s ID number, lab ID number, date, time and method of collection and transported to the laboratory following the World Health Organization (WHO) guidelines. Whereas, those samples which were not properly labelled, improperly transported with visible signs of contamination and lacked patients’ complete information were excluded.

### Isolation and identification of bacteria

All the samples were inoculated directly into blood agar, MacConkey agar and chocolate agar. *P. aeruginosa* colonies were identified on the basis of colony characteristics on the respective media. Colonies showing typical *P. aeruginosa* characteristics on culture and morphology on gram staining were transferred to nutrient agar and incubated at 37 °C for 24 h. Further, identification was done by pyocyanin (blue-green) pigmentation and conventional biochemical tests including catalase and oxidase test. Similarly, *P. aeruginosa* were separated from other *Pseudomonas* spp by observing growth on cetrimide agar at 42 °C for 24 h [13].

### Antibiotic susceptibility testing

Antibiotic susceptibility tests of all isolates were performed using Kirby Bauer disc diffusion method on Mueller-Hinton Agar with recommended antibiotics by CLSI 2020 guidelines [14].The antibiotics used were gentamicin (GEN,30 µg), amikacin (AK, 10 µg), ciprofloxacin (CIP, 5 µg), ceftazidime (CAZ, 30 µg), cefepime (CPM, 30 µg), aztreonam (AT, 30 µg), imipenem (IPM, 10 µg), piperacillin (PI,30 µg), piperacillin-tazobactam (PIT), meropenem (MRP, 10 µg), ofloxacin (OF, 30 µg), Levofloxacin (LEV, 30 µg) and colistin (CL,10 µg) from Hi-Media, Laboratories Pvt. Ltd. India.

Isolates that were non-susceptible to at least one agent in ≥ 3 antimicrobial categories have been categorized under MDR [15].

### Screening for MBL producers

All the multidrug resistant *P. aeruginosa* were subjected for MBL detection. Phenotypic confirmatory test for MBL producers were carried out by using Imipenem-EDTA combined disc diffusion test (CDDT).

Two imipenem discs were placed on agar plate’s containing lawn culture of test organism. 10 µl of EDTA solution was applied to one of the imipenem disc, placed 25 mm apart (center-center) and the plate was incubated at 37 °C. After 18–24 h of incubation, an increase of ≥ 7 mm in the zone diameter of imipenem-EDTA disc as compared to imipenem disc alone was considered to be positive test for the presence of MBL [14].

### Determination of minimum inhibitory concentration of colistin by broth dilution method

Colistin resistance was phenotypically detected by broth microdilution method, using colistin sulphate powder (Sigma- Aldrich). The results of MIC were interpreted according to European Committee on Antimicrobial Susceptibility Testing guidelines (EUCAST) [16].

### DNA extraction and quantification

**Fig. 1:**
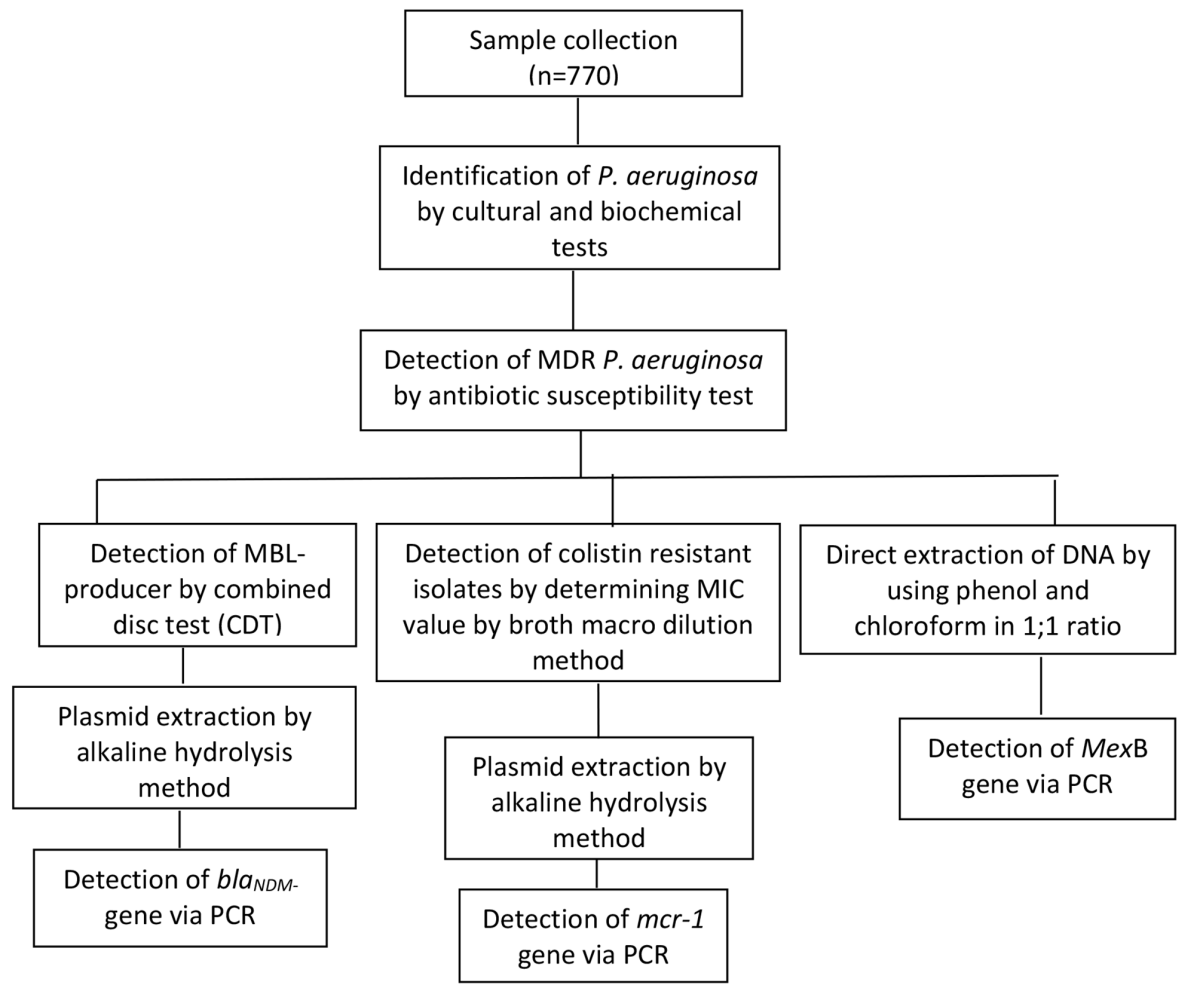
Flowchart of the procedure

For molecular analysis, all MDR *P. aeruginosa* isolates were subjected to the alkaline lysis method for plasmid DNA extraction and phenol chloroform method for chromosomal DNA extraction [17]. The extracted DNA was quantified by using Nanodrop and its band was visualized on agarose gel stained with ethidium bromide (Fig. 1).

### PCR analysis of ***bla***_***NDM−1***,_***mcr-1***, and ***MexB*** genes

PCR of *bla*_NDM−1_, *mcr-1* and *Mex*B genes were performed as below in the table.

**Table:** 

Genes	Primer	Base pair	PCR reaction mixture	Temperature profile and Reference
***Bla***_***NDM−1***_	** F =** GGT TTG GCG ATC TGG TTT TC R=(CGG AAT GGC TCA TCA CGA TC	**621 bp**	8.5 µl of 2X master mix, 0.5 µl of 10 picomolar primer (forward and reverse), 12.5 µl of nuclease free water and 3 µl of extracted DNA template	initial denaturation at 94 °C for 5 min, followed by 36 cycles of 95 °C for 30 s, 52 °C for 40 s and 72 °C for 50 s with final extension at 72 °C for 5 min [18].
***Mcr-1***	** F =** CGGTCAGTCCGTTTGTTC **R =** CTTGGTCGGTCTGTAGGG	**309 bp**	21 µl 1X master mix, 0.5 µl of 10 pmolar primer (forward and reverse) and 3 µl of extracted plasmid DNA	Initial heating at 95 °C for 15 min, then 35 cycles of 94 °C for 10 s, 57 °C for 90 s and 72 °C for 90 s and final extension at 72 °C for 10 min. [19]
**MexB**	**F =** TGTCGAAGTTTTTCATTGATAG **R =** AAGGTCAC GGTGATGGT	**280 bp**	21 µl of 1X Qiagen master mix, 0.5 µl of 10 pmole primers (forward and reverse) and 3 µl of extracted DNA template.	Initial heating at 94 °C for 3 min, then 32 cycles of 94 °C for 30 s, 55 °C for 45 s and 72 °C for 1 min and final extension at 72 °C for 7 min [20].

### Data analysis

Data obtained were analyzed using SPSS version 18. The p < 0.05 was considered statistically significant.

## Results

### Growth profile in different clinical samples

Out of total 770 different clinical samples cultured during the study, bacterial growth was observed in 27.4% (n = 211) samples. *P. aeruginosa* was isolated only from 4.6% (n = 36) samples. Majority of *P. aeruginosa* isolates were obtained from tracheal aspirates (36.1%), followed by sputum (25%) and blood (13.9%).

### Antibiotic susceptibility pattern

Altogether 36 isolates of *P. aeruginosa* were tested against different antibiotics classes. Among these antibiotics tested more number of *P. aeruginosa* were found susceptible towards carbapenems and piperacillin + β-lactam inhibitor (66.7%) antibiotic classes (Table 1). Likewise, about 50% of *P. aeruginosa* isolates were found to be multidrug resistant.

**Table 1:** Antibiotic susceptibility pattern of *P. aeruginosa*

Antibiotic category	Antibiotics	Antibiotics susceptibility pattern	
		Sensitive (%)	Resistant (%)
Penicillin	Piperacillin	18(50)	18(50)
Penicillin + β-lactam inhibitor	Piperacillin + tazobactam	24(66.7)	12(33.3%)
3rd generation cephalosporin	Ceftazidime	18(50)	18(50)
4th generation cephalosporin	Cefepime	18(50)	18(50)
Monobactams	Aztreonam	17(47.2)	19(52.8)
Carbapenems	Imipenem	24(66.7)	12(33.3)
	Meropenem	24(66.7)	12(33.3)
Aminoglycosides	Amikacin	18(50)	18(50)
	Gentamicin	18(50)	18(50)
Fluroquinolones	Ciprofloxacin	19(52.8)	17(47.2)
	Levofloxacin	17(47.2)	19(52.8)
	Ofloxacin	17(47.2)	19(52.8)

### Metallo-β-lactamase detection

Using CDDT phenotypic method to identify metallo-β-lactamase (MBL), the prevalence of MBL producing *P. aeruginosa* isolates was found to be 33.3%. There was statistical significant association between MBL producer and MDR isolates (p < 0.001) (Table 2).

**Table 2:** Distribution of MBL producers among MDR isolates

		Metallo-β-lactamase		Total	p-value (Fischer’s exact test)
		MBL producer (%)	MBL non-producer (%)		
Multidrug resistant	MDR	12(66.7)	6(33.3)	18	
	Non-MDR	0(0.00)	18(100)	18	0.000
		12	24	36	

### Determination of MIC value of colistin

Out of 36 isolates of *P. aeruginosa*, 2 (11.2%) were found to colistin resistant. MICs of colistin for *P. aeruginosa* isolates were found to be ranged between 1(µg/ml) to 8(µg/ml). Highest MIC value was found to be 8 µg/ml (Table 3).

**Table 3:** MIC value of colistin among *P. aeruginosa* isolates

Organism	Number	Concentration of colistin			
		**1 µg/ml**	**2 µg/ml**	**4 µg/ml**	**8 µg/ml**
*P. aeruginosa*	36	28 (77.8%)	6 (16.7%)	0	2 (5.6%)

### Prevalence of ***NDM-1*** among multidrug resistant ***P. aeruginosa***

**Fig. 2:**
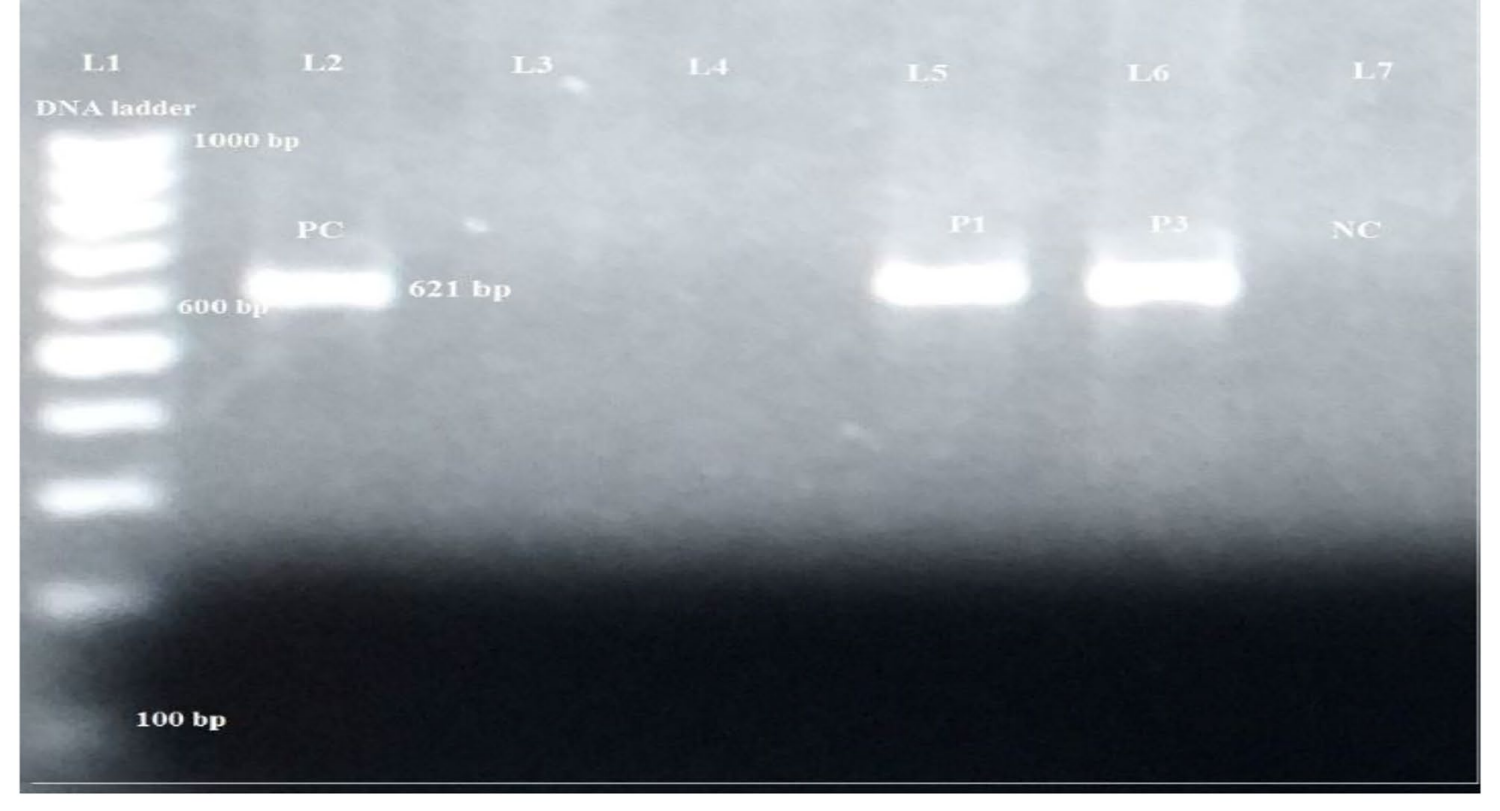
PCR amplification of *bla*_*NDM−1*_ gene in MDR *P. aeruginosa* isolates

New Delhi metallo β-lactamase-1 (NDM-1) genotypes were detected by conventional PCR method using *bla*_*NDM−1*_ specific primer pair. Among 18 MDR *P. aeruginosa*, 3 (16.7%) isolates were *bla*_*NDM−1*_ positive. Similarly, 25% of MBL producer harboured *bla*_*NDM−1*_ (Fig. 2).

Lane L1: DNA size marker (100–1000 bp); Lane 2: positive control; Lane 3&4: *bla*_*NDM−1*_negative; Lane 5&6: *bla*_NDM−1_ positive; Lane 7: negative control.

### Characteristics of ***bla***_***NDM−1***_ harbouring ***P. aeruginosa*** isolates

Three *P. aeruginosa*, P1, P3 and P6 were isolated from tracheal swab, urine and pus respectively, were found to be harbouring *bla*_*NDM−1*_ genes. All three isolates were susceptible to colistin and were isolated from ICU ward patients (Table 4).

**Table 4:** Characteristics of *bla*_*NDM−1*_ harbouring *P. aeruginosa* isolates

Characteristics	Patient-1	Patient-2	Patient 3
Isolate Number	P1	P3	P6
Age(yr)/Sex	57y/Female	59y/Male	30y/Female
Hospital location	ICU	ICU	ICU
Specimen source	Tracheal swab	Urine	Pus
Underlying disease/diagnosis	Sub-dural haemorrhage	Post stroke seizure	TB meningitis, transverse myelitis and communicating hydrocephalus
Co-morbid conditions	Diabetes& hypertension	Diabetes& hypertension	None
Antimicrobials used prior to detection of *bla*_*NDM−1*_	Cefotaxime, gentamicin and ciprofloxacin	Flucloxacillin, meropenem and ciprofloxacin	Nitrofurantoin, ceftriaxone and flucloxacillin
**Antibiotics tested**			
Amikacin	R	R	R
Gentamicin	R	R	R
Ciprofloxacin	R	R	R
Imipenem	R	R	R
Meropenem	R	R	R
Piperacillin	R	R	R
Piperacillin/tazobactam	R	R	R
Aztreonam	R	R	R
Ofloxacin	R	R	R
Levofloxacin	R	R	R
Ceftazidime	R	R	R
Cefepime	R	R	R
Colistin	S	S	S

### Prevalence of ***mcr-1*** among multidrug resistant ***P. aeruginosa***

**Fig. 3:**
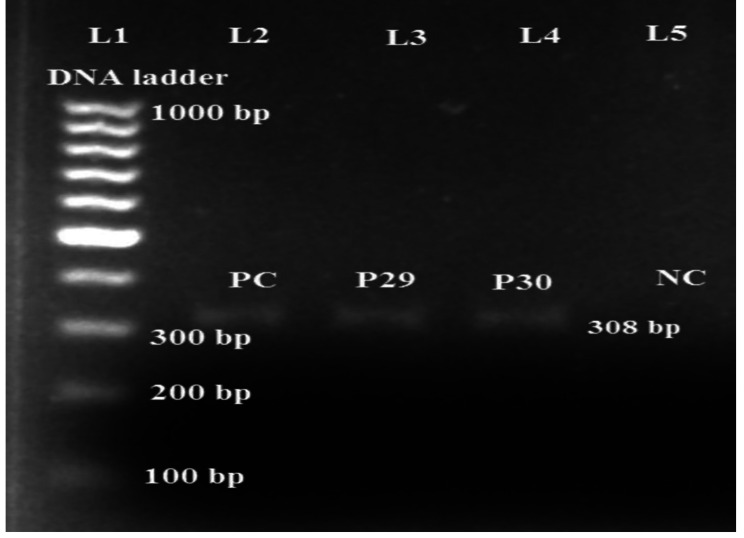
PCR amplification of *mcr-1* gene in MDR *P. aeruginosa* isolates

*mcr-1* gene was detected by conventional PCR method using *mcr-1* specific primer pair. Among 18 MDR *P. aeruginosa*, 2 (11.1%) were *mcr-1* positive (Fig. 3).

Lane L1: DNA size marker (100–1000 bp); Lane 2: positive control (*mcr-1* positive plasmid DNA); Lane 3&4: *mcr-1* positive; Lane 5: negative control.

### Prevalence of ***MexB*** among multidrug resistant ***P. aeruginosa***

**Fig. 4:**
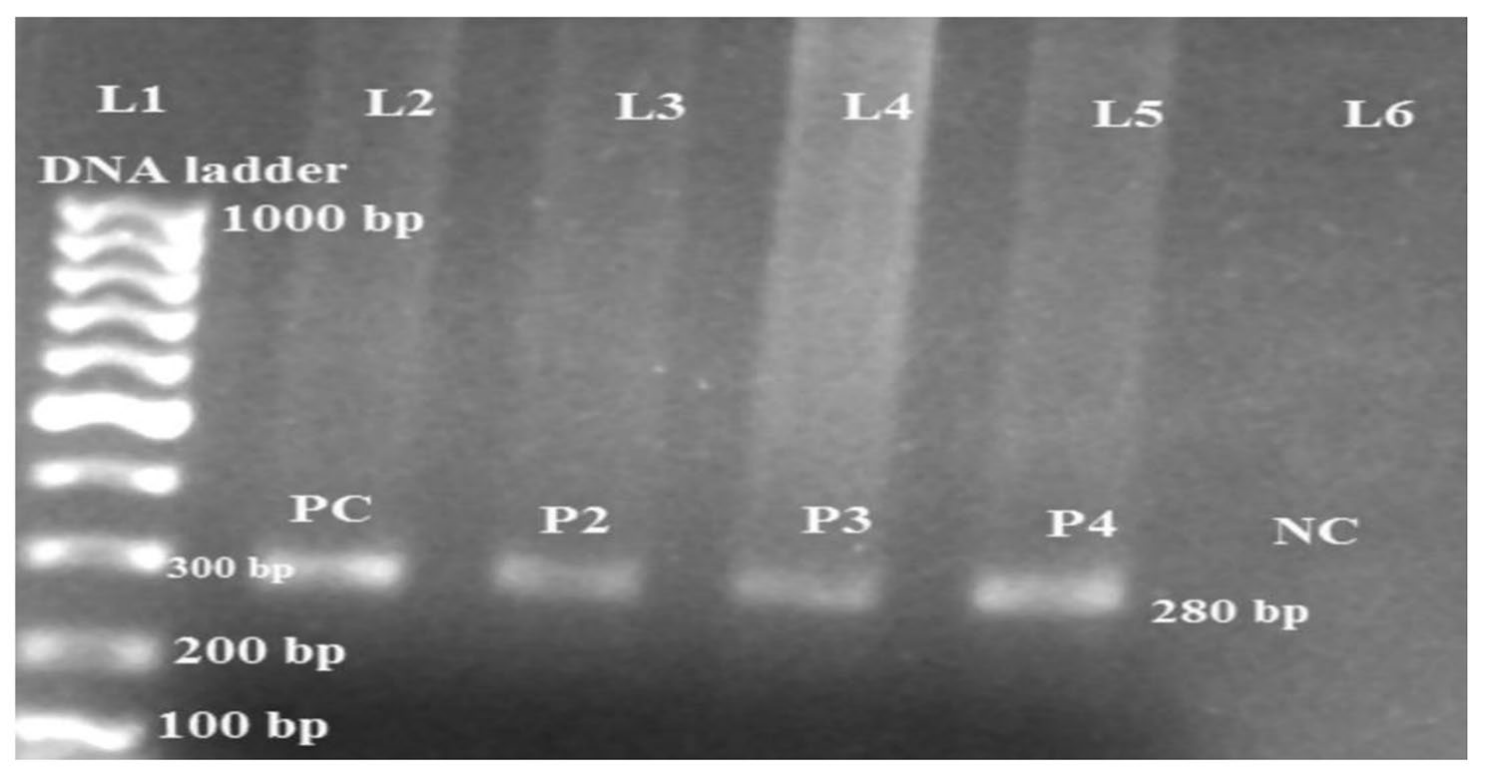
PCR amplification of *MexB* gene in MDR *P. aeruginosa*

Among 18 MDR *P. aeruginosa*, 17 (94.4%) isolates were *MexB* positive (Fig. 4).

Lane L1: DNA size marker (100–1000 bp); Lane 2: positive control; Lane 3/4/5: *MexB* positive; Lane 6: negative control.

## Discussion

In this study, a total of 36 *P. aeruginosa* were isolated and identified from various clinical specimens. 50% of them were found to be MDR and 33.3% were MBL-producer. Among MDR isolates 16.7%, 11.1% and 94.4% of them were found to be harbouring *bla*_*NDM−1*,_*mcr-1* and *MexB* genes respectively.

According to our study, prevalence rate of *P. aeruginosa* was found to be 4.6%. Variable prevalence rate of *P. aeruginosa* has been reported from previous studies done in Nepal that ranges from 2.2 to 17.05% [21–23]. Such variations in result might be due to different factors such as; differences in samples, different microbiological methodologies used and variation in geographical location [24]. Furthermore, in our study, highest number of *P. aeruginosa* were isolated from respiratory samples followed by blood. This result was found similar to the findings of previous studies conducted in Nepal [25, 26]. Therefore, our findings infers that *P. aeruginosa* is one of the predominant organism to cause respiratory tract infections. *P. aeruginosa* are developing resistance to different anti-pseudomonal drugs via various mechanisms that is resulting them to develop into most dangerous and dreaded bug [22, 27]. In our study, half of the total isolates were found to be multidrug resistant. Such high rates of MDR *P. aeruginosa* were also reported in previous studies from Nepal that ranges from 50 to 55.5% [25, 27].

Although carbapenem are considered as a drug of choice to treat *P. aeruginosa* infections, the emergence of carbapenem resistance significantly decreases its usefulness [8]. In our study, 33.3% of *P. aeruginosa* isolates were found to be MBL producer. This finding was found similar to the previous study conducted in Nepal [28]. However, some studies in other countries have recorded various percentages of MBL-producing *P. aeruginosa* such as 38.3% in Brazil, 47.3% in Taiwan, 62% in Greece, 53.4% in Italy, 69.8% in India and 68.7% in Egypt [29–32]. Furthermore, it has also been found that among 18 MDR *P. aeruginosa*, 12 isolates were MBL producers and their association was statistically significant (p < 0.001).

Among MBL types, detection of *bla*_*NDM−1*_ producer is alarming to clinical settings because presence of *bla*_*NDM−1*_ increases fear of diseases in future not to be cured by antibiotics [9]. In our study, we found that among MBL positive isolates, 25% of them carried *bla*_*NDM−1*_ gene. This result was found higher than the previous study conducted in Nepal [7]. Therefore, our result infers that prevalence of *bla*_*NDM−1*_ harbouring *P. aeruginosa* is in increasing trend in Nepal. So, periodic detection of carbapenem resistant isolates is necessary in order to implement new antibiotic treatment policies. Furthermore, in our study, it has been found that, these *bla*_*NDM−1*_ positive *P. aeruginosa* isolates were from the patients of intensive care unit (ICU) from tracheal swab, urine and pus sample and were resistant to all antibiotics tested except colistin. Therefore, this outcome suggests that colistin should be kept as the reserve drug to treat MDR/XDR *P. aeruginosa*. In addition, as *bla*_*NDM−1*_ positive *P. aeruginosa* were detected from ICU patients, treatment of them should be done separately because *bla*_*NDM−1*_ is known as rapidly spreading gene that can spread swiftly [7]. Further, the detail clinical characteristics of the three patients harbouring *bla*_*NDM−1*_ positive *P. aeruginosa* are depicted in Table 3.

Colistin is an oldest antibiotic that has been re-included in the list of useful antibiotics as a ˈreserve drugˈ to treat *P. aeruginosa* infection. However, resistance to colistin is also increasing recently [5]. In our study, 5.6% of isolates were found to be resistant to colistin with MIC value of 8 µg/ml. This finding differs from those reported from Pakistan [2]. Furthermore, it has been found that all colistin resistant isolates were from ICU patients and were *mcr-1* positive. Therefore, our finding indicates that colistin resistant *mcr-1*gene is spreading across the world which is a global challenge for therapeutic option to treat emerging pathogens in future.

To our knowledge, there exists no report on emergence of plasmid mediated *mcr-1* in *P. aeruginosa* in Nepal. Though reports of *mcr-1* in *P. aeruginosa* in Nepal are not reported till the date, knowledge of its prevalence is important because *P. aeruginosa* is a ubiquitous organism with high colonization capacity and ability to survive for a long period of time in the hospital settings. In this study, we found that the *mcr-1* harbouring isolates were from patients admitted to ICU ward and were resistant to all available antibiotics tested. Therefore, early detection and isolation of patients harbouring superbugs should be done to prevent its further spread.

Multidrug resistance (MDR) pumps play an important role in the antibiotic resistance of *P. aeruginosa*. MexAB-OprM is the most important efflux pump, overexpression of it plays a significant role in development of MDR strains [33]. To our knowledge, there is no previous report of detection of *MexB* gene in *P. aeruginosa* in Nepal. In our study, among MDR *P. aeruginosa* isolates, 94.4% of them were found to be harbouring *MexB* gene. This finding is in accordance with the previous study [34]. Detection of *MexB* in almost all MDR isolates infers that overexpression of MexAB efflux pump might be helping isolates to develop into MDR by pumping antibiotics outside of the cell. Therefore, *MexB* is also an important antimicrobial resistant biomarker that might help to trace MDR isolates.

### Limitations of the study

This study was unable to detect other resistance mechanisms that might also helping *P. aeruginosa* develop into MDR due to limited resources. Also, this study was conducted for a limited time period on the patients of a single hospital which does not provide the overall scenario of antibiotic resistance of Nepal.

## Conclusion

To the best of our knowledge, this study is first to report about the presence of *mcr-1* and *MexB* genes in *P. aeruginosa* isolates in context of Nepal. Furthermore, detection of *bla*_*NDM−1*_ and *mcr-1* in a highly mobile genetic element is a major highlight of our study because of its global threat to antimicrobial therapy that is leading them to evolve into deadest pathogen ‘Superbug’. Therefore, continuous screening and monitoring of phenotypic as well as genotypic phenomenon of resistance in *P. aeruginosa* are necessary to trace such superbugs. Furthermore, timely control of these resistant pathogens could help to prevent further spread.

## Electronic supplementary material

Below is the link to the electronic supplementary material.

Supplementary Material 1

## Data Availability

The data used to support the findings are available from the corresponding author upon request.
