# Supplementary material for: Detection of blaNDM−1,mcr-1 and MexB in multidrug resistant Pseudomonas aeruginosa isolated from clinical specimens in a tertiary care hospital of Nepal
Source: BMC Microbiol. 2023 May 25;23:153. doi: 10.1186/s12866-023-02906-w (PMC10210380; doi:10.1186/s12866-023-02906-w)
Supplement: Supplementary file 1 — Supplementary Material 1 [file 12866_2023_2906_MOESM1_ESM.docx]

Sample collection (n=770)

Identification of *P. aeruginosa* by cultural and biochemical tests

Detection of MDR *P. aeruginosa* by antibiotic susceptibility test

Detection of MBL-producer by combined disc test (CDT)

Detection of colistin resistant isolates by determining MIC value by broth macro dilution method

Direct extraction of DNA by using phenol and chloroform in 1;1 ratio

Plasmid extraction by alkaline hydrolysis method

Plasmid extraction by alkaline hydrolysis method

Detection of *Mex*B gene via PCR

Detection of *bla_NDM-_* gene via PCR

Detection of *mcr-1* gene via PCR

**Figure 1: Flowchart of the procedure**


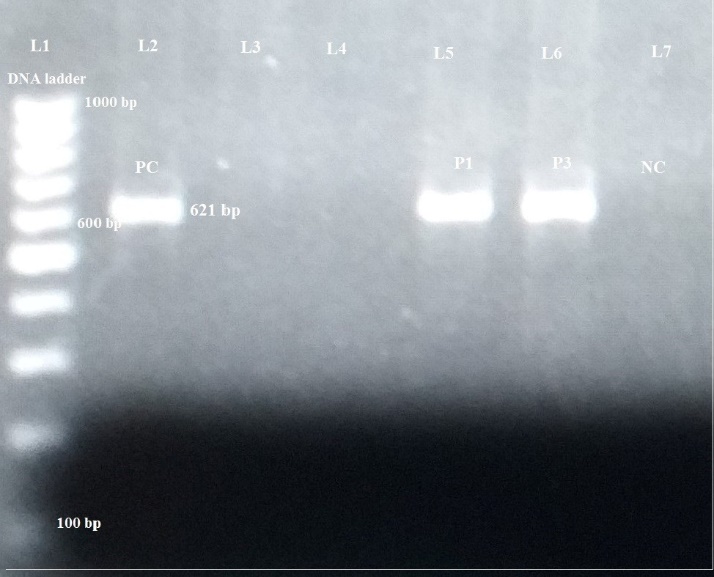


**Figure 2: PCR amplification of *bla_NDM-1_* gene in MDR *P.aeruginosa* isolates**

Lane L1: DNA size marker (100-1000 bp); Lane 2: positive control; Lane 3&4: *bla_NDM-1_* negative isolates; Lane 5&6: *bla*_NDM-1_ positive isolates; Lane 7: negative control

**
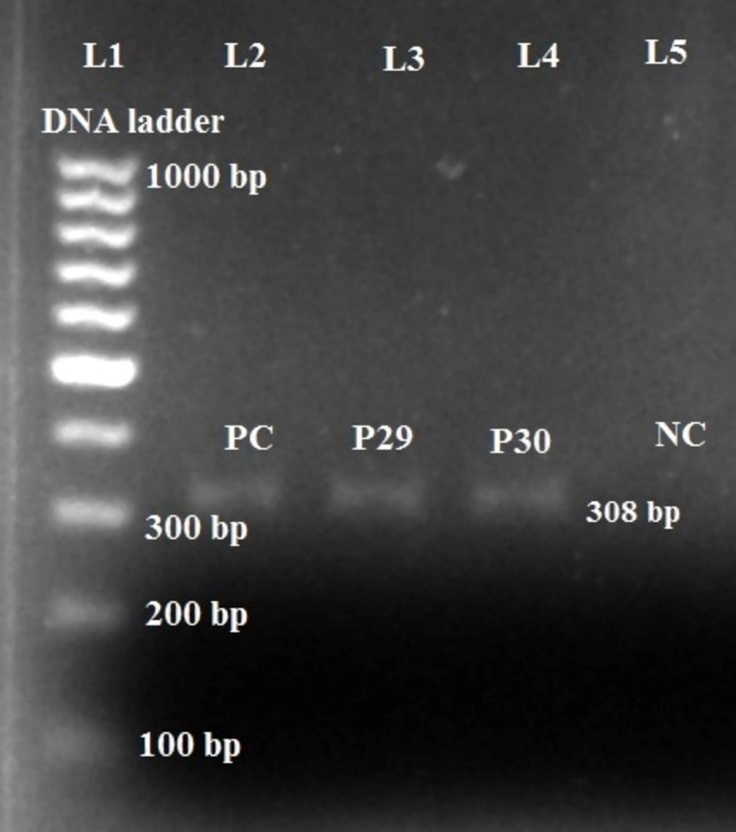
**

**Figure 3: PCR amplification of *mcr-1* gene in MDR *P. aeruginosa* isolates**

Lane L1: DNA size marker (100-1000 bp); Lane 2: positive control (*mcr-1* positive plasmid DNA); Lane 3&4: *mcr-1* positive isolates; Lane 5: negative control


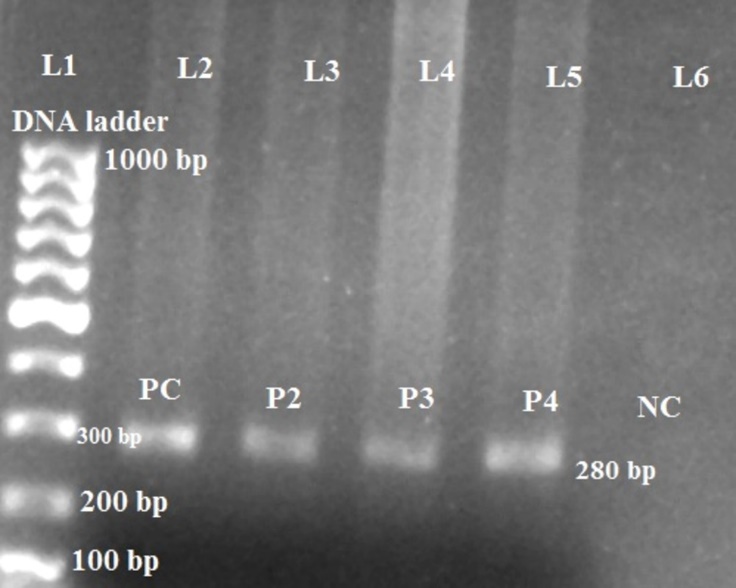


**Figure 4: PCR amplification of *MexB* gene in MDR *P. aeruginosa***

Lane L1: DNA size marker (100-1000 bp); Lane 2: positive control; Lane 3/4/5: *MexB* positive isolates; Lane 6: negative control.

100 bp
